# Fire or Frost in Medicine?

**Published:** 1897-03-20

**Authors:** 


### The Hospital

A Journal of

The Medical Sciences and Hospital Administration,

Vol. XXI.—No. 547.] [March 20, 1897.

### Fire or Frost in Medicine?

It has been said that the chief difference between the mind of Shakespeare and that of many other great intellects is that Shakespeare's was a mind of living fire and light, whereas the mind of the ordinary considerable thinker is a mind of light and frost. We have come to understand in our time the value of fire, as well as of light; its enkindling, vivifying, purifying value. The modern medical mind, it is often contended, though undoubtedly a mind of light, is yet of light without fire, without heat; and, therefore, of frigid, feeble, and often ineffectual vitality and power. The man of science —and the man of medicine is nothing if not a man of science—too often thinks it an occasion for self-congratulation that he has extinguished feeling in himself. He speaks of "the dry light of science," and holds himself to be an absolutely typical instance of it, with unmixed pride and inner satisfaction.

Well, ours are practical times, practical above all things; and the fairest scientific theory which was ever propounded finds its way to the medical lumber-room in five years if there is no living soul of practice in it. What the medical man has to think of is, for himself, how he may be most largely successful at the bedside; and, for his profession, how he may make the whole of medicine as largely serviceable to the happiness and progress of man as he can. And, if these be his aims, we contend that the temper of mind with which he pursues them is a factor of prime importance both for the individual doctor himself and for his profession. It is not too much to say that the spirit in which a man sets himself to his work is the most important of all the factors which determine his success or his failure.

When the doctor cultivates the cold temper, the frigid tone, the dry light, the doctor is wrong; or if the doctor be right Shakespeare was wrong. In science, work and intelligence make conquests undoubtedly. If the work and the intelligence be there, without enthusiasm, some conquests may certainly be made. But if the work and intelligence be present, with enthusiasm, the conquests will be both much greater and much more widely effectual. As modern examples of men of science in whom enthusiasm has prevailed may be mentioned Huxley, Lord Lister, Lord Kelvin, Pasteur. Has the science of these men lost anything because they have been men of eager and strenuous emotions, men who have not striven to exclude feeling from their lives and work? It has gained immensely; but the world has gained from it immensely more.

Modern medicine needs warmth, fire, the "enthusiasm of humanity," as Sir John Seeley called it. Tennyson reminded us that "knowledge comes, but wisdom lingers." In medicine "knowledge comes" amply, abundantly, overwhelmingly; but practice, successful practice, "lingers." There cannot be a doubt of it. What is the reason? The aged among us are not now ignorant. On the contrary, they have in most instances these great advantages over their younger brethren that they possess both the old knowledge and the new, with a wide personal experience to help both the one and the other. They can well afford, therefore, to suppress both jealousy and envy in themselves. But it has happened that they have inherited a suspicious and watchful temperament from their unscientific predecessors; and so they turn upon all things which may happen to be a little different from those to which their professional fathers accustomed them, looks of cold aversion and dislike. But the new must and will have its day. New ideas are apt to kindle enthusiasm in those upon whom they lay hold; and medicine must not only reconcile itself to, but must welcome enthusiasm.

Frost means winter and death; and that quite as much in the minds and ways of men as in the world of vegetation and animal life. An old profession, like an old country, must always be specially on its guard against rigid rules and customs which cannot yield. Every decaying generation groans at the flippancy of its incoming successor. But that is merely comical Up to the present time, at any rate, each new generation—in medicine especially—has displaced something old, and brought in many things new, to the inconceivable advantage both of the profession and the world. And is it likely that wisdom and science will die with the present generation of seniors?

A passion for effectiveness—that is the chief essential for a successful doctor and for a progressive medicine. Medical men, the presidents of our colleges, the lecturers in our class-rooms, the busy consultant, and the popular general practitioner need, in addition to their knowledge and experience, the fire and warmth which made Shakespeare the greatest of all Englishmen. English medicine ought to be great, as England herself is great. England is great by virtue of the two things which made Shakespeare great, living fire and light, working out to practical ends. Medicine has the light; let her, without fear or hesitation, lay hold of the living fire, and she, too, will be great with a greatness like that of Shakespeare, which for three centuries, charged with revolutions infinite in number and variety, has shown not the slightest sign of waning.

